# Recurrences after Pulsed Field Ablation of Atrial Fibrillation: Incidence, Mechanisms, Predictors, and Comparison with Thermal Energy

**DOI:** 10.3390/medicina60050817

**Published:** 2024-05-16

**Authors:** Riccardo Vio, Enrico Forlin, Paolo China

**Affiliations:** 1 Department of Cardiothoracic, Vascular Medicine and Intensive Care, Dell’Angelo Hospital, 30174 Mestre-Venice, Italy; enrico.forlin.1@studenti.unipd.it (E.F.); paolo.china@aulss3.veneto.it (P.C.); 2 Department of Cardiac, Thoracic and Vascular Sciences and Public Health, University of Padua, 35128 Padua, Italy

**Keywords:** atrial fibrillation, catheter ablation, pulsed field ablation

## Abstract

Pulsed Field Ablation (PFA) is the latest and most intriguing technology for catheter ablation of atrial fibrillation, due to its capability to generate irreversible and cardiomyocytes-selective electroporation of cell membranes by delivering microsecond-lasting high-voltage electrical fields, leading to high expectations. The first trials to assess the clinical success of PFA, reported an arrhythmia-free survival at 1-year of 78.5%, while other trials showed less enthusiastic results: 66.2% in paroxysmal and 55.1% in persistent AF. Nevertheless, real world data are encouraging. The isolation of pulmonary veins with PFA is easily achieved with 100% acute success. Systematic invasive remapping showed a high prevalence of durable pulmonary vein isolation at 75 and 90 days (range 84–96%), which were significatively lower in redo procedures (64.3%). The advent of PFA is prompting a reconsideration of the role of the autonomic nervous system in AF ablation, as PFA-related sparing of the ganglionated plexi could lead to the still undetermined effect on late arrhythmias’ recurrences. Moreover, a new concept of a blanking period could be formulated with PFA, according to its different mechanism of myocardial injury, with less inflammation and less chronic fibrosis. Finally, in this review, we also compare PFA with thermal energy.

## 1. Introduction

Pulsed field ablation (PFA) is the latest most commercially available technology for catheter ablation of atrial fibrillation. In contrast to conventional techniques (i.e., radiofrequency and cryoablation) based on thermal energy, PFA lesions are created by delivering microsecond-lasting high-voltage electrical fields, which cause irreversible electroporation of cell membranes [1].

Preclinical studies showing the irreversible modality of cellular death associated with PFA—limiting pulmonary vein (PV) reconnections—together with selectivity for cardiomyocytes (potentially sparing surrounding structures responsible for some procedure-related complications) created high expectations for this new technique. A computational comparison showed that, for equivalent lesion depths, PFA, compared to radiofrequency (RF), generates wider and more symmetrical lesions in the cardiac chambers [2].

While safety benefits were confirmed also in human studies, with no esophageal fistula or permanent phrenic palsy, the long-term efficacy of PFA remains to be determined as recurrences may occur even several years post-ablation [3].

In this review, we gathered the mounting evidence regarding recurrences after PFA, focusing on their incidence, underlying mechanisms, and predictors. Preliminary data comparing PFA outcome on arrhythmia-free survival versus conventional technologies using thermal energies are analyzed. 

## 2. Incidence of Recurrences after PFA

Data on the follow-up after PFA are reported by first trials and newer real-world data. The first trials to assess the clinical success of PFA were IMPULSE (NCT03700385), PEFCAT (NCT03714178), and PEFCAT II (NCT04170621) trials. By pooling together these trials, the 1-year outcome after PFA was calculated [4]. The authors reported an arrhythmia-free survival at 1-year for the entire cohort of 78.5%.

Recently, longer follow-up data of this cohort of patients were released [5]. The median follow-up duration was 49 months; at this point, 73% of patients remained free from atrial arrhythmias and 68% were free from AF or atrial flutter without Class I/III antiarrhythmic drugs. Patients who did not experience recurrences during the first year remained free from late recurrence in 89% of cases.

However, the protocol of IMPULSE, PEFCAT, and PEFCAT II trials included invasive remapping at three months with the reisolation of reconnected pulmonary veins. Therefore, we can speculate that the long-term outcome without this early invasive strategy would have been worse. Nonetheless, after the 1-year follow-up there were no prespecified and scheduled monitorings and freedom from recurrences might have been overestimated as acknowledged by the authors [5].

The PULSED AF trial showed less enthusiastic results, reporting an efficacy at 1 year of 66.2% in paroxysmal AF and 55.1% in persistent AF [6].

However, real-world data are encouraging. Ruwald et al. recently reported, over a mean follow-up of 308 days in a 121-patient court, that 18.2% of the cases experienced clinically significant recurrences or required initiation of anti-arrhythmic drugs [7]. The Kaplan–Meier event-free estimate at 365 days was 80% (88% for paroxysmal AF and 69% for persistent AF).

The MANIFEST-PF is a multi-national registry that showed a 1-year Kaplan–Meier estimate for freedom from atrial arrhythmia of 78.1% [8].

### How to Assess Recurrences

Different methods were used to assess recurrences after PFA in the available studies. The best way to assess recurrences is by using an implantable loop recorder, able to detect subclicinal arrhythmias [9]. Prolonged ECG-holter monitoring (of at least 7 days) at prespecified time points (e.g., 3, 6, and 12 months post-ablation) is a feasible alternative.

In the study by Reddy et al., weekly transtelephonic ECG together with 72-hour ECG-Holter monitoring at 6 and 12 months were performed [4]. The nearly identical same strategy was adopted also in the PULSED AF trial [6].

Longer ECG-Holter recordings were adopted by Maurhofer et al. who used 7-day ECG-Holter monitoring at 3, 6, and 12 months to detect possible recurrences, although this intensive strategy did not lead to a higher percentage of reported recurrences [10].

## 3. Underlying Mechanisms of Recurrences

### 3.1. Pulmonary Veins’ Reconnection 

The cornerstone of catheter ablation for AF is the isolation of pulmonary veins [11]. This target can be easily achieved with PFA with eight standard applications for each vein. Procedural time is low and acute success is 100%. This was reported by Reddy et al. and later confirmed by real-world data [12,13].

According to a systematic review and meta-analysis, durable pulmonary vein isolation is associated with a lower risk of recurrences after catheter ablation. However, pulmonary reconnection is common even in AF-free patients, affecting up to 58% of them [14].

Preclinical models using PFA showed 100% lesion durability at 30 days [15]. Sites ablated with PFA showed a loss of myocardial fibers with fibrocellular replacement, neovascularization, and neocollagen deposition.

The PersAFOne study was specifically designed for assessing pulmonary veins’ isolation durability after PFA in persistent AF patients [12]. An outstanding 96% of pulmonary vein isolation was found at 75-day invasive remapping after the first procedure, with 100% of the left atrial posterior walls isolated that remained durable ablated.

Systematic invasive remapping was also performed at 90 days in another study. In the optimized PFA waveform, cohort pulmonary vein isolation durability was 96% and patients with durable pulmonary vein isolation were 84% [4].

Other evidence comes from electroanatomical mapping findings at redo procedures. Among patients scheduled for redo procedures for clinically relevant recurrences after PFA, more than one third showed durable pulmonary vein isolation [16]. The remaining patients (64.3%) showed from one to three pulmonary veins reconnected. 

These data are in contrast with the ones reported in another study that analyzed findings from repeat ablation using high-density mapping. In fact, Tohoku et al. found a remarkably low reconnection rate (only 9.1% of pulmonary veins were reconnected) [17]. However, we can speculate that this rate might have been higher if all patients with atrial tachycardia after PFA had been scheduled for redo (they selected 25/46 patients with recurrences, targeting preferable patients with atrial tachycardia rather than atrial fibrillation). 

#### Pulmonary Vein Reconnection Pattern 

Conflicting results were reported by recent studies. Right pulmonary veins were found to be more reconnected than their left counterparts at redo procedures by Ruwald et al. [18]. Gaps were localized particularly at the right anterior carina. The authors speculated that these findings may be explained by numerous factors: higher PFA catheter displacement inside right pulmonary veins because of diaphragm contraction; tendency of the catheter to orientate more posteriorly because of the anatomy of transeptal puncture; and difficult positioning of the catheter in the right inferior pulmonary vein. 

Magni et al. confirmed this tendency, and in its case series pulmonary vein reconnections were mostly localized at the right carina, anterior aspect of the right superior pulmonary vein (RSPV), and inferior aspect of the right inferior pulmonary vein (RIPV) [16]. Gaps in left pulmonary veins were localized mainly in the posterior part of the left superior pulmonary vein (LSPV) and postero-inferior part of left inferior pulmonary vein (LIPV). In details, the percentage of reconnection per single pulmonary vein was 50% for RIPV, 35.7% for LIPV, 28.6% for LSPV and 21.4% for RSPV. Again, the authors underscored the lower maneuverability of the PFA catheter in the right inferior pulmonary vein and the higher myocardial thickness in the above-mentioned areas. 

In contrast with these results, Gunawardene et al. reported that superior pulmonary veins are more reconnected than their inferior counterparts [19]. Other authors reported the left superior pulmonary vein to be the more reconnected, pointing out its complex ostium anatomy because of its strict relationship with the left atrial appendage [17].

Knowing the most frequent reconnection patterns may guide operators to optimize energy delivery in such areas, which can be achieved by using intracardiac echocardiography (ICE) (see below—paragraph “Section 4.2 Technical aspects”).

Table 1 summarizes the most frequent reconnection sites of pulmonary veins after PFA according to the abovementioned studies.

At present, there is still not enough evidence to define a common pattern of pulmonary vein reconnection after PFA and future studies are needed in this field.

### 3.2. Antral Lesion

Another factor that influences the incidence of recurrences after catheter ablation of AF is how pulmonary vein isolation is achieved. Wide antral isolation has been proved to be more effective than ostial pulmonary vein isolation [20]. The rationale for a better outcome by ablating a larger area at the junction of pulmonary veins with the left atrium, is the removal of electrophysiological heterogeneity that is capable of promoting micro-reentry that might maintain AF [21,22,23,24,25].

The other traditional advantage of wide antral pulmonary vein isolation, which is the avoidance of pulmonary vein stenosis, is not relevant for PFA as this is not a possible complication of the procedure [26,27].

Blockhaus et al. compared the size of the acute antral lesion between PFA and cryoablation by using electroanatomical mapping of the left atrium. They found that lesions created by PFA were more antral (antral lesion size of PFA vs. cryoballoon 67.03 ± 12.69% vs. 57.39 ± 10.91%, respectively, *p* = 0.01) [28]. The authors called for further clinical trials to prove if these results may correlate with better clinical otucomes. 

Conflicting evidence was reported by another group who studied instead chronic antral lesion size at invasive remapping (performed at day 75 per protocol in the PFA cohort or at the time of redo procedure for the thermal cohort) [29]. According to the study results, PFA creates similar areas of isolation compared to thermal energy (radiofrequency or cryoablation or laser balloon).

However, lesions created by PFA could be large enough to unintentionally ablate and isolate the left atrial posterior wall. In fact, there are early reports of unexpected fused posterior wall lesions after PFA [30].

If not completey isolated, the left atrial posterior wall may paradoxically favor recurrences after PFA, by creating a perfect corridor and substrate for atypical atrial flutters—see below [17].

### 3.3. Atrial Arrhythmia Responsible for Recurrences 

Recurrences after catheter ablation of AF may be caused by AF itself, atypical atrial flutters, or atrial tachycardia. Recurreces in the first 3 months post-ablation (the so-called blanking period) are traditionally not considered as true recurrences, as they can represent a transient phenomenon and may not occur in longer follow-ups. 

Among the available literature, all studies are in agreement that the main arrhythmia responsible for recurrences after PFA is AF.

Kueffer et al. reported that the major arrhythmia responsible for recurrences after PFA is AF (62% of cases), followed by atypical left atrial flutter (28%) and atrial tachycardia (10%) [31]. Similar results were reported by other authors, with 65% of recurrences caused by AF and 35% caused by regular atrial tachycardias [18]. In the study by Magni et al., 85% of patients with recurrences showed AF (alone or in combination with atrial flutter), and the remaining 15% of patients had atrial flutter or atrial tachycardia only [16].

Meanwhile, Tohoku et al. found 25/45 (55%) of patients with recurrences with AF and the remaining 20/45 (45%) with atrial tachycardias whose critical ishtmus was mainly located in the posterior wall in a narrow corridor of viable myocardium in between the lesions created for pulmonary vein isolation [17].

**Figure 1:**
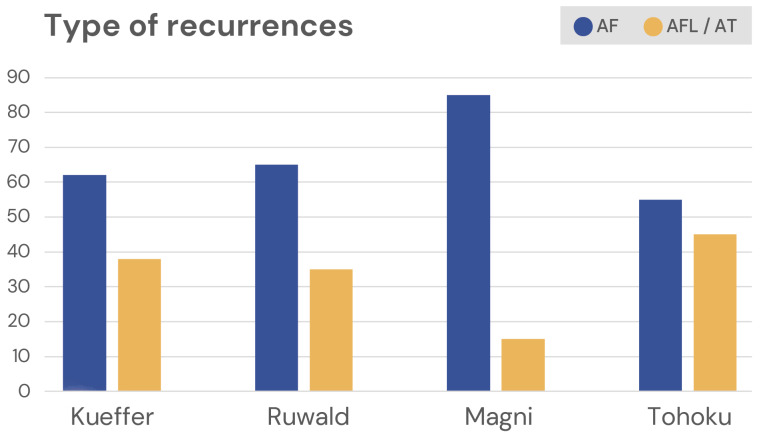
Arrhythmias responsible for recurrences after PFA ablation. AF = atrial fibrillation; AFL = atrial flutter; and AT = atrial tachycardia. Kueffer [31]; Ruwald [18]; Magni [16]; Tohoku [17].

Figure 1 graphically shows the arrhythmias rensponsible for recurrences after PFA.

Even though AF is the major arrhythmia for recurrences, atrial tachycardias are still responsible for a sizeable part of procedural failures, and they could in part be avoided by an optimized procedural workflow. Patients with predominantly atrial tachycardia as the arrhythmia responsible for recurrence usually show durable isolation of the pulmonary veins [18]. Electroanatomical mapping immediately after PFA in the index procedure may allow us to identify incomplete lesions in the posterior wall, which deserve additional applications to completely suppress the region. Another possibility suggested by some authors is the empirical isolation of the posterior wall in all patients, in order to avoid the eventual creation of arrhythmogenic substrate for reentry and atypical atrial flutters [31]. Refinement of the procedural workflow and its impact in the success of the ablation remain to be investigated. 

### 3.4. Autonomic Function

The cardiac nervous system plays a critical role in AF initiation and maintenance [32,33]. The key structures of the cardiac autonomic nervous system are the ganglionated plexi, which surround the atria in the epicardium. Given the proximity of ganglionated plexi with the ostium of pulmonary veins and the non-selective transmural lesions, conventional thermal energy may create bystander damage to such structures. Some studies correlated the thermal energy-induced damage of the ganglionated plexi and the resulting autonomic changes with the long-term success of AF ablation [34].

In preclinical studies PFA energy has been showed to be selective for the myocardium, sparing neighboring structures such as nerves (i.e., phrenic nerve) [35,36]. In the clinical setting, researchers have investigated if PFA might also preserve ganglionated plexi and their autonomic function.

By using an extracardiac vagal stimulation, Stojadinovic and colleagues demonstrated a significantly lower responsiveness of both sinoatrial and atrioventricular nodes in patients treated with radiofrequency compared to those treated with PFA [37].

Furthermore, other studies reported that, compared to PFA, thermal energy significantly increases the heart rate after ablation, confirming the marginal effect on the autonomic nervous system of PFA [38]. The preservation of ganglionated plexi has been elegantly demonstrated in the same study: vagal effects were reproduced by the stimulation of the vast majority (71%) of the ganglionated plexi located inside the low voltage (ablated) areas. Conversely, high-frequency stimulation in ganglionated plexi after radiofrequency resulted in either an abolished (18/20) or reduced (2/20) vagal effect. 

Del Monte et al. interestingly reported that intraprocedural vagal reactions are more frequent during PFA than radiofrequency ablation (70% vs. 28%, *p* = 0.001) [39]. Nonetheless, the acute suppression of vagal response after PFA recovered almost completely in 10 min. The authors hypothesized that this phenomenon might be explained by the high-voltage pulsed field energy causing an extreme stimulation of the ganglionated plexi, leading to a transient modulation of their function for a temporary depletion of parasympathetic neurotransmitters.

Serum S100 is a recognized denervation marker, and it has been found that it increases far less after PFA compared to cryoballool ablation [40]. The magnitude of increase in S100 blood levels was not related to transient bradycardia, excluding a possible causality between autonomic reaction and S100 increase during PFA. Autonomic responses during PFA are probably related to the high-frequency stimulation of the ganglionated plexi. 

Whether the preserved activity of ganglionated plexi after PFA will result in higher late recurrences (>2 years postablation) remains to be determined. However, lower autonomic involvement with PFA could be useful to prevent potentially negative long-term effects like inappropriate sinus tachycardia or decrese in exertion capacity. This is supported by histological and functional preclinical evidence in animal models, and clinically by recent studies showing preserved heart rate variability after PFA [38,39,40,41].

### 3.5. Non-Pulmonary Vein Triggers

Pulmonary veins’ isolation has been the cornerstone of AF ablation procedure since the discovery of their central role in AF initiation and maintenance. However, particularly in AF persistence or in the presence of structural heart diseases, structural and electrophysiological modifications of the atria are induced, leading to the onset of non-pulmonary vein triggers responsible for AF recurrences after pulmonary vein isolation [42]. Nevertheless, available data are conflicting: in most randomized trials extensive ablation strategies failed to show any benefit over the sole pulmonary vein isolation (CAPLA trial, DECAAF II trial). In the LIBERATION trial, however, persistent AF patients were randomized to PV isolation or extensive ablation strategy (including PVs, LA septum, coronary sinus, and left posterior wall), showing in the latter group statistically significant higher rates of freedom from atrial tachyarrhythmias (20%, 15%, 10% at 1-, 2- and 3-year follow-ups in PV only isolation and 65%, 50%, 40% in extensive ablation strategy, respectively, *p* < 0.001) [43].

The left atrium posterior wall (LAPW) is one of the most frequent targets for an adjunctive ablation strategy. Pulmonary veins and LAPW indeed share a common histological and embryological derivation and spontaneous ectopic trigger activity from LAPW, as has been previously reported [44,45,46,47]. Furthermore, as has already been said, LAPW may generate a corridor for reentrant arrhythmias after pulmonary vein isolation, which is especially in persistent AF. Performing a homogeneous LAPW isolation may, however, be tough with thermal energy, considering the relatively small area of erogation and the proximity to the esophagus, site of potentially serious adverse events, and thus it often limits thermal energy delivery [48]. In this context, PFA could be a valid alternative due to the larger erogation area and myocardial tissue selectivity, allowing the generation of complete and homogeneous lesions. Feasibility and safety of LAPW isolation with PFA was first assessed in the PersAFOne study [12]. Gunawardene et al., in a real-world comparative study on persistent AF patients, showed favorable outcomes with Kaplan estimates for freedom from atrial arrhythmias at a 500-day follow-up of 82% among patients with index LAPW isolation in addition to pulmonary vein isolation versus 73% for patients with the sole pulmonary vein isolation. No major complications occurred [49]. Efficacy and durability of LAPW isolation with PFA was also highlighted by Kueffer et al., which found an 85% rate of durable LAPW isolation at redo procedures in patients with atrial arrhythmia recurrence [50]. However, other studies showed no benefit in arrhythmia-free survival in AF patients treated with PFA with additional isolation of LAPW compared to their counterparts treated with PV isolation alone [51].

Among the other non-pulmonary vein triggers, there are pioneering experiences with PFA of superior vena cava (SVC). SVC ablation with thermal energy sources has been proven to be effective in reducing AF recurrence in selected patients [52,53]. The feasibility and safety of PFA of SVC has been assessed in animals by Zhu et al. and then, firstly, reported in humans by Tao et al., in a paroxysmal AF patient with arrhythmia recurrence despite pulmonary vein isolation [54,55].

Isolation with RF of left atrial appendage may provide additional benefits, expecially in patients with persistent AF (even though there are concerns for the pro-thrombotic risk associated) [56]. In canine models, PFA was successful in isolating the left atrial appendage [57]. Whether these results may be safely and effectively replicated in humans remains to be determined.

Other studies are required to evaluate the safety and efficacy of PFA of non-PV triggers, in particular exploiting its specificity for the myocardium, which could maybe highlight some benefits in the isolation of these structures, avoiding damage to neighboring tissues (e.g., esophagus during LAPW ablation and phrenic nerve during SVC ablation). This advantage is limited when it comes to ablate areas adjacent to coronary vessels, as PFA deliveries have been reported to provoke coronary artery spasm, which sometimes persists after erogations and requires nitrates administration to end the process [58].

## 4. Predictor of Recurrences

### 4.1. Characteristics of Patients

Several factors are associated with recurrences after PFA. As already demonstrated with the use of thermal energy, among the most important ones seems to be the pattern of arrhythmia before ablation (paroxysmal vs. persistent). In the study by Ruwald et al., patients with paroxysmal AF had significantly lower recurrences compared to their persistent counterparts (AF-free survival 88% vs. 69%, *p* = 0.001) [7]. Other authors quantified the risk conferred by the presence of persistent AF in a 39% increase in the risk of recurrences (HR 1.39, *p* < 0.001) [8]. Moreover, older age plays a significant role in the risk of recurrence (HR for age > 65 yo = 1.57), together with atrial enlargement (HR for LA diameter > 45 mm = 1.33) and longer procedural time (HR for procedural time > 60 min = 1.30). A normal left ventricular ejection fraction was associated with a better outcome of the ablation with fewer recurrences (HR for EF > 50% = 0.78). 

In the European EU-PORIA registry, another two variables were independently associated with higher recurrences after PFA ablation: CHA2DS2-VaSc score and body mass index (OR 1.034 and 1.154, respectively) [59].

Overall, the above-mentioned factors have been extensively studied also with AF ablation with conventional thermal energy, reporting similar results [60]. This is somewhat expected, being factors related to patients’ characteristics rather than procedural aspects related to a new technology (i.e., PFA).

### 4.2. Technical Aspects

From the very first studies, ICE has emerged as a useful tool for several purposes during the AF ablation procedure. Its importance was firstly assessed with RF ablation and has been also highlighted in the recent 2024 EHRA expert consensus statement on catheter and surgical ablation of atrial fibrillation: observational studies and meta-analyses associated ICE use with reduction in procedure and fluoroscopy time, complication rates, repeat ablation, in-hospital mortality, and hospitalization length [61,62,63,64,65]. These achievements have been linked to the possibility of guiding energy delivery, especially in areas with high risks of complications, like that adjacent to the esophagus or within the PVs [66]. The usefulness of ICE has been confirmed in PFA, in which it was also able to increase the effectiveness of ablation by enhancing the durability of pulmonary vein isolation (PV durability 98.2% with ICE vs. 91.8% without ICE, *p* = 0.022) [4]. The authors correlated this finding with the facilitation of catheter positioning with ICE, which eventually leads to better pulmonary isolation.

Data from the study by Kueffer et al. showed that both the 35 mm PFA catheter and the use of a voltage amplitude of 1.9 kV were associated with the need for a redo procedure [31]. The lower performance of the 35 mm catheter was also reported in another study, in which it was related with an increased pulmonary vein reconnection rate [17]. It has been speculated that spreading the same electrical field over a larger area could reduce the effectiveness of the ablation. However, the results might be confounded by the fact that the 35 mm catheter is frequently used in patients with enlarged atria, another classical factor associated with recurrences (see the above paragraph). Finally, a voltage of 2.0 kV is now recommended by the catheter manufacturer and therefore lower voltages (e.g., 1.9 kV or 1.8 kV) should be avoided.

### 4.3. Early Recurrences during the Blanking Period

Data on thermal ablation suggest that, during the blanking period (i.e., traditionally considered to be the first three months post-ablation), the occurrence of atrial tachyarrhythmias might not imply a failure of the interventional therapy, even though it is associated with late recurrences [67,68,69]. Recently, researchers have also focused on the role of early recurrences during the blanking period following PFA. They found that early recurrences happen in approximately 1/5 of patients following PFA, tripling the risk of late recurrences (hazard ratio 3.370, *p* < 0.001) [70]. Interestingly, they reported no difference in the rate of late recurrences in patients experiencing early recurrences in the first 45 days, compared to those having early recurrences between 45 and 90 days post-ablation. Such a finding differs from thermal ablation, where very early (<45 days) recurrences are more likely to result in long-term ablation success compared to those occurring in the second part of the blanking period [68,69]. The different mechanism of myocardial injury related to PFA (with less inflammation and less chronic fibrosis) could be responsible for a different significance of early recurrences and the whole concept of the blanking period could be reformulated in PFA.

Recently, the blanking period has been shortened from 3 months to 8 weeks by EHRA expert consensus guidelines [65]. The reason for this choice mainly lies in the different impact of early recurrences in patients treated with RF ablation on the risk of long-term AF relapse. In fact, if the absence of early recurrences during the blanking period is known to be associated with a high likelihood with freedom from late recurrences, the positive predictive value of early recurrences is variable [71]. Data from several studies and trials show that the risk of long-term AF relapse grows as the early recurrences occur later in the first 3 months [69,72,73,74]. For example, in the ADVICE trial, freedom from AF at the 12-month follow-up was 77.2% in patients without recurrences in the first 3 months and 62.6%, 36.4%, and 7.8% in patients experiencing recurrences in the first, second and third month post-ablation, respectively [68]. According to this evidence, a consensus among experts decided to reduce the classical blanking period to the above-mentioned 8 weeks, in order to reduce the misclassification of patients with early recurrences [65].

## 5. Comparison with Thermal Energy 

PFA’s main advantages rely on its high myocardial tissue specificity, sparing non-cardiac cells and thus theoretically avoiding a large part of the complications related to the indiscriminate delivery of thermal energy in radiofrequency and cryothermal ablation. 

The ADVENT trial was the first multicenter, randomized controlled trial comparing the safety and effectiveness of PFA to thermal energy source ablation for the treatment of drug-resistant paroxysmal AF. In this trial, PFA met the criteria for noninferiority according to effectiveness (with a success rate of 73.3% and 71.3% in the PFA and thermal cohort, respectively) and safety (with an occurrence of a composite of serious adverse events of 2.1% and 1.5% in the PFA and thermal cohort, respectively). The criteria for superiority in effectiveness were not met. PFA was however superior for the secondary safety endpoint regarding reduction in pulmonary vein cross-sectional area from baseline to 90 days (3.3% and 19.5% in the PFA and thermal cohort, respectively). Moreover, results showed lower procedural times but a longer fluoroscopic time with PFA [75].

These data were confirmed by the recent meta-analysis from Aldaas et. al, including a total poll of 1012 AF patients evaluated in six different comparative studies: between PFA and thermal energy groups no statistically significant differences were seen in periprocedural adverse events (RR 1.20, 95% CI 0.59–2.44) or atrial arrhythmias recurrences (RR 0.64, 95% CI 0.31–1.34), but with statistically shorter procedure times (mean difference −21.95 min, *p* = 0.0003) and longer fluoroscopy times (mean difference +5.71 min, *p* = 0.01) in the PFA group [76]. The authors speculated that the lack of differences in periprocedural complications, despite the theorized safety advantages of PFA, could be partly explained by operator inexperience, partial PFA waveforms optimization, and the adoption of preventive maneuvers in the thermal energy cohort [76].

Larger trials are thus required to the evaluate safety and effectiveness of PFA compared to thermal energy source ablation. In this regard, the SINGLE SHOT CHAMPION (NCT05534581) and BEAT AF (NCT05159492) will provide additional evidence on this topic. Future studies should also investigate the impact of PFA on left atrial stiffness, which has been associated with increased left atrial pressure and worsening post-ablation diastolic function in AF patients treated with RF [77].

## 6. Conclusions

PFA has the potential to open a new era in AF ablation. Larger studies are needed to confirm the effectiveness of PFA in the long term and to prove its theorical superiority in safety.

## Figures and Tables

**Table 1:** Pulmonary vein reconnection patterns after PFA ablation according to the literature.

	RSPV	Right Carina	RIPV	LSPV	LA Ridge	Left Carina	LIPV
Ruwald [18]	X	X	X				
Magni [16]	X	X	X				
Gunawarde [19]	X			X			
Tohoku [17]				X	X		

LA = left atrial; LIPV = left inferior pulmonary vein; LSPV = left superior pulmonary vein; RIPV = right inferior pulmonary vein; and RSPV = right superior pulmonary vein.

## Data Availability

Not applicable.
